# Acupuncture Attenuates Renal Sympathetic Activity and Blood Pressure via Beta-Adrenergic Receptors in Spontaneously Hypertensive Rats

**DOI:** 10.1155/2017/8696402

**Published:** 2017-02-08

**Authors:** Jing-Wen Yang, Yang Ye, Xue-Rui Wang, Fang Li, Ling-Yong Xiao, Guang-Xia Shi, Cun-Zhi Liu

**Affiliations:** ^1^Acupuncture and Moxibustion Department, Beijing Hospital of Traditional Chinese Medicine Affiliated to Capital Medical University, 23 Meishuguanhou Street, Dongcheng District, Beijing 100010, China; ^2^Beijing University of Chinese Medicine, 11 Beisanhuan East Road, Chaoyang District, Beijing 100029, China

## Abstract

The sympathetic nervous system, via epinephrine and norepinephrine, regulates *β*-adrenergic receptor (*β*-AR) expression, and renal sympathetic activation causes sustained increases in blood pressure by enhanced renin release. In this study, we aim to investigate the effect and underlying mechanism of acupuncture at Taichong (LR3) on renal sympathetic activity in spontaneously hypertensive rats. Unanesthetized rats were subject to daily acupuncture for 2 weeks. Mean blood pressure (MBP) and heart rate variability (HRV) were monitored at days 0, 7, and 14 by radiotelemetry. After euthanasia on the 14th day, blood and the kidneys were collected and subject to the following analyses. Epinephrine and norepinephrine were detected by ELISA. The expression of *β*-ARs was studied by western blotting and PCR. The renin content was analyzed by radioimmunoassay. 14-day acupuncture significantly attenuates the increase of MBP. The HRV indices, the standard deviation of all normal NN intervals (SDNN), and the ratio of the low-frequency component to the high-frequency component (LF/HF) were improved following acupuncture. Renal sympathetic activation induced upregulation of epinephrine, norepinephrine, and renin content were attenuated by acupuncture. In addition, acupuncture decreased *β*1-AR expression and improved *β*2-AR expression. These results indicated that acupuncture relieves the increased MBP via the regulation of renal sympathetic activity and *β*-ARs.

## 1. Introduction

Enhanced sympathetic nervous system activity is linked to the development and maintenance of hypertension [1–3]. The extensive sympathetic innervation of the kidney occupies a special place in the development of hypertension. Calaresu and Ciriello [4, 5] showed that renal afferent nerves project directly into a number of areas in the central nervous system, such as the lateral tegmental fields, the paramedial reticular nucleus, the dorsal vagal complex of the medulla, and the lateral hypothalamic area, contributing to arterial blood pressure regulation. Renal efferent sympathetic activity participates in renin release, sodium retention, and reduced renal blood flow, which contribute to the development of hypertension [6–8]. In animal models, Kumagai and his colleagues [9] found that stimulation of afferent sympathetic nerve increased systemic sympathetic nerves activity and caused vasoconstriction. Moreover, the attenuation of either the efferent or afferent renal nerves theoretically contributes to lowering blood pressure (BP) [8]. The renal sympathetic nervous system regulates BP mainly through catecholamines (epinephrine and norepinephrine) binding to *β*-adrenergic receptors (*β*-ARs). Moreover, *β*1-ARs in the kidney may activate renin release [10] and, consequently, result in the renin-angiotensin-aldosterone system (RAAS) activation and angiotensin II formation followed by vasoconstriction [11]. Therefore, the renal sympathetic nervous system and *β*-ARs are critical for regulating peripheral resistance and play important roles in the development of hypertension.

Acupuncture has been recommended as a complementary therapy for hypertension. In 1996, 64 acupuncture indications were declared by World Health Organization in Milan conference including hypertension [12]. Numerous animal and clinical studies have reported the efficacy of acupuncture in treating hypertension [13–15]. Although there is some disagreement among the reports, the majority of them indicate that acupuncture causes a significant decrease in BP [13]. However, the underlying mechanism through which acupuncture lowers BP remains to be elucidated. Acupuncture could affect the sympathetic nervous system. Knardahl et al. found that acupuncture produces moderate hypoalgesia in humans paralleled by a regulation of muscle sympathetic nerve activity [16]. Song et al. suggested that acupuncture pretreatment improved the survival rate in rats with lethal endotoxemia, which involves the activation of the autonomic nervous system [17]. Research has shown that electroacupuncture modulates the renal sympathetic nerve activity in chronic kidney disease rats [18]. Although the relationship between acupuncture and the sympathetic nervous system has been demonstrated in a variety of models, the antihypertensive effect of acupuncture on spontaneously hypertensive rats (SHRs) via sympathetic nervous system and *β*-ARs remains unclear.

The present study was conducted to examine the hypothesis that the activation of the renal sympathetic nervous system in SHRs is mediated by acupuncture. Furthermore, we assessed if this renal nerve-mediated effect contributes to the downregulation of *β*1-AR and upregulation of *β*2-AR, leading to the decreased BP in SHRs.

## 2. Materials and Methods

### 2.1. Experimental Animal

Male SHR and WKY rats (12 weeks), weighing 260–300 g, were purchased from Vital River Laboratory Animal Technology Co. Ltd (Beijing China). The animals were housed in cages at 22 ± 2°C and humidity of 40 ± 5% under a 12-hour light/dark cycle and received standard diet and water ad libitum. The experimental procedures were in accordance with the Guidelines for the Institutional Animal Care and Use Committee of China Academy of Chinese Medical Sciences (Beijing, China).

### 2.2. Animal Grouping and Acupuncture Stimulation

The rats were randomly divided into 4 groups, the control group (WKY), the model group (SHR), the acupuncture stimulation group at LR3 (located between the first and second metatarsal bones on the dorsum of the foot) (Acu), and nonacupoint acupuncture stimulation (Non-Acu), with 10 rats in each group. Sterilized disposable stainless steel needles (0.3 mm × 40 mm, Hwato, China) were inserted 5 mm deep at LR3. For the Non-Acu group, the rats received similar treatment as Acu group but the acupuncture site was at a sham acupoint (on the bilateral hypochondrium, 10 mm above iliac crest) to replace LR3. The rats in the WKY and SHR groups were given the same time and same level catching-grasping stimulus without acupuncture stimulation. Acupuncture proceeded for 30 seconds each time, once daily for a period of 2 weeks (1 day rest after 6 days of treatment).

### 2.3. Radiotelemetry

We measured BP and HRV by radiotelemetry combined with fast Fourier transform analysis of BP and HRV. DSCF-FS01 HRV devices or DSCF-FS02 BP devices (Softron, USA) were implanted in rats to follow BP and HRV changes continuously over time. The telemetric techniques and the techniques employed to analyze the autonomic nervous system are described in detail elsewhere [19]. Briefly, we anesthetized the rats with intraperitoneal injection of pentobarbital sodium (Sigma, USA). Then, The telemeter was placed in the abdominal cavity, the blood pressure catheter tip was inserted into the descending abdominal aorta, and the electrode leads approximated the Lead II orientation as previously described [20]. The rats recovered for 7 days before baseline BP and HRV values were recorded. By this time, the rats had regained their circadian BP and HRV rhythm; and surgery and anesthesia-induced changes had abated.

Data were sampled every 5 min for 10 sec continuously day and night and stored on a hard disk. BP and HRV were recorded using the SP 2006 Lan software. Continuous beat-by-beat values of BP and HRV were recorded during morning hours. Measurements to analyze the autonomic control of BP and HR were performed between 8:00 and 10:00 AM.

### 2.4. ELISA

NE and E contents in the plasma and kidney were measured by enzyme-linked immunosorbent assay (ELISA) kit (R&D Systems, USA) according to the manufacturer's instruction. Absorbance in each well was measured using microplate reader (Thermo Fisher, USA) at 450 nm. Concentrations of NE and E in the plasma and renal were determined by interpolation from the standard curve.

### 2.5. Real-Time Quantitation PCR

Total RNA was extracted from the kidney using Trizol Reagent (Invitrogen) according to the manufacturer's instructions. Quantification of the relative amounts of mRNA was performed using the Rotor-Gene6000 1.7 version software (Corbett research). In brief, 2 *μ*L of the cDNA was mixed with primers, 100 nM of probe, and 1x TaqMan Universal Master Mix in each reaction. Predeveloped assay reagents were used and were purchased from Applied Biosystems. Sequence for the primers and TaqMan probes for the rat *β*1-AR, *β*2-AR, and renin are shown in Table 1. Samples were tested in triplicate, and differences of threshold cycles between target genes and house-keeping gene (18s rRNA) were calculated. The relative mRNA abundance in the treatment groups was calculated using 2^−ΔΔCT^ method using the control group as the calibrator according to the manufacturer's user manual. The value of relative mRNA quantity for control group is 1 with arbitrary units.

### 2.6. Western Blot Analysis

Total protein in each sample was measured by the BCA assay (Bio-Rad, USA) using bovine serum albumin as the standard protein. A fixed amount of protein (20 *μ*g) from each sample was fractionated by 10% SDS-polyacrylamide gel electrophoresis (SDS-PAGE) and transferred to a polyvinylidene difluoride (PVDF) membrane (Pall, USA). Membranes were incubated in 5% milk in TBS for 1 h in room temperature. For identification of *β*-ARs, membranes were exposed to primary antibodies (anti-*β*1-AR, 1 : 250 dilution, or anti-*β*2-AR, 1 : 500 dilution, abcam, USA). After washing, the membranes were incubated with secondary antibodies (HRP-conjugated anti-rabbit antibody, 1 : 5000 dilution, KBL, USA). The bound antibodies were detected using the enhanced chemiluminescent reagent (GE Health Care, USA). Data are presented as the *β*-ARs to actin ratio and then expressed as fold-change compared to WKY group.

### 2.7. Radioimmunoassay

For determination of PRC, 50 *μ*L of blood was collected into EDTA-containing 75-*μ*L microhematocrit tubes from conscious rats by tail vein puncture. For determination of renal renin content, samples of kidney cortex were dissected under the microscopy, frozen in liquid nitrogen, and stored at −80°C until assay. For renin analysis, tissue was weighed, homogenized with two 30-second pulses in a 100-fold excess of homogenization buffer (5% [vol/vol] glycerol, 0.1 mmol/L of PMSF, 10 mmol/L of EDTA, and 0.1 mmol/L of 4-[2-aminomethyl]benzenesulfonyl fluoride) using a Polytron homogenizer (Kinematica), and centrifuged at 4°C at 140,000*g* for 5 minutes. Supernatants were incubated with saturating concentrations of rat renin substrate, and angiotensin I generation was assayed by radioimmunoassay (DiaSorin).

### 2.8. Statistical Analysis

Data analysis was performed with SPSS software version 16.0. All values were expressed as the mean ± standard error (SEM). Comparison between the treatment group and the control group was performed by one-way ANOVA test followed by a post hoc least significant difference multiple comparison test. A *P* value < 0.05 was considered statistically significant.

## 3. Results

### 3.1. Effect of Acupuncture on BP in SHRs

**Figure 1:**
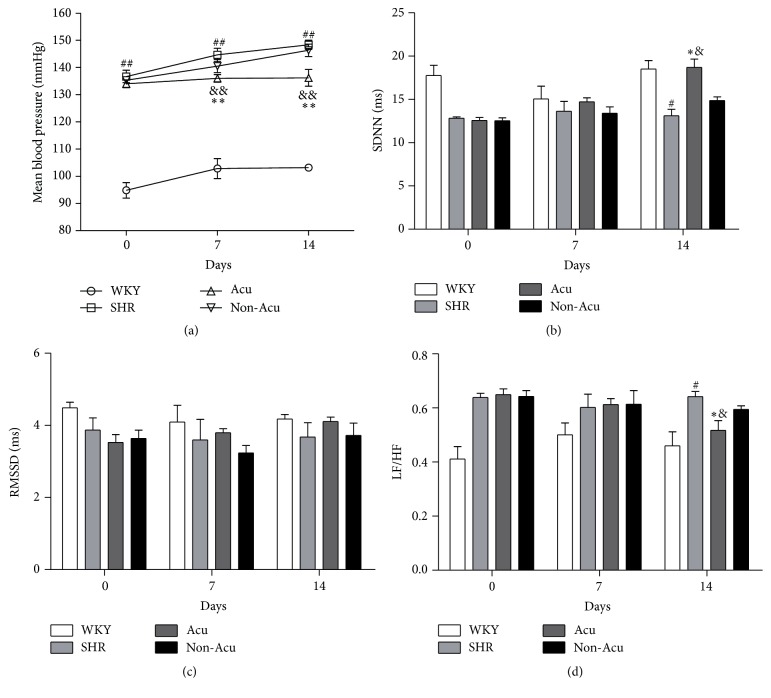
Effects of acupuncture on mean blood pressure (a) and heart rate variability (b–d) as measured by radiotelemetry in all groups. Data are presented as mean ± SEM (*n* = 10 rats). # and ## indicate *P* < 0.05 and *P* < 0.01, respectively, compared with the WKY group; *∗* and *∗∗* indicate *P* < 0.05 and *P* < 0.01, respectively, compared with SHR group; & and && indicate *P* < 0.05 and *P* < 0.01, respectively, compared with Non-Acu group.

To confirm the efficacy of acupuncture, BP levels were measured in all groups by the telemetry method after two weeks of acupuncture treatment (Figure 1(a)). Before acupuncture, the mean blood pressure (MBP) of SHR, Acu, and Non-Acu group were insignificantly different (*P* > 0.05), but significantly higher than in the WKY rats. After 7 days of acupuncture, the MBP was significantly decreased compared with SHR group and Non-Acu group (*P* < 0.01), and this decrease was sustained throughout the treatment period (days 7–14). Although the MBP of Acu group was significantly decreased, it was still higher than the WKY group (*P* < 0.01) over the experimental period. These results suggested that acupuncture could lower BP but is unable to reduce it to normal levels.

### 3.2. Effect of Acupuncture on the Balance of Autonomic Nervous in SHRs

To investigate whether the attenuation of BP in SHRs by the treatment with acupuncture is associated with beneficial effects of the automatic nervous system, we used radiotelemetry method to examine the heart rate variability (HRV) in all groups at days 0, 7, and 14 (Figures 1(b)–1(d)). The values of standard deviation of all normal NN intervals (SDNN) and root mean square of differences between adjacent NN intervals (rMSDD) show acupuncture effects on HRV by mean variation of the short-term period. Before acupuncture, the SDNN of the WKY group was higher than the other groups, and SDNN was slightly improved in the Acu group after 7 days of treatment without significance (*P* > 0.05). In the 14 days of acupuncture, the SDNN was significantly increased compared with the SHR group and Non-Acu group (*P* < 0.01). However, there was no significant change in RMSSD among the four groups (*P* > 0.05).

The ratio of the low-frequency component to the high-frequency component (LF/HF) shows acupuncture effects on autonomic regulation. LF/HF of the SHR group, Acu group, and Non-Acu group were significantly higher than that of the WKY group at days 0 and 7 (*P* > 0.05). On the 14th day, acupuncture significantly reduced the ratio of LF/HF compared with the SHR group and Non-Acu group (*P* < 0.01).

### 3.3. Effect of Acupuncture on the Contents of Catecholamine in SHRs

**Figure 2:**
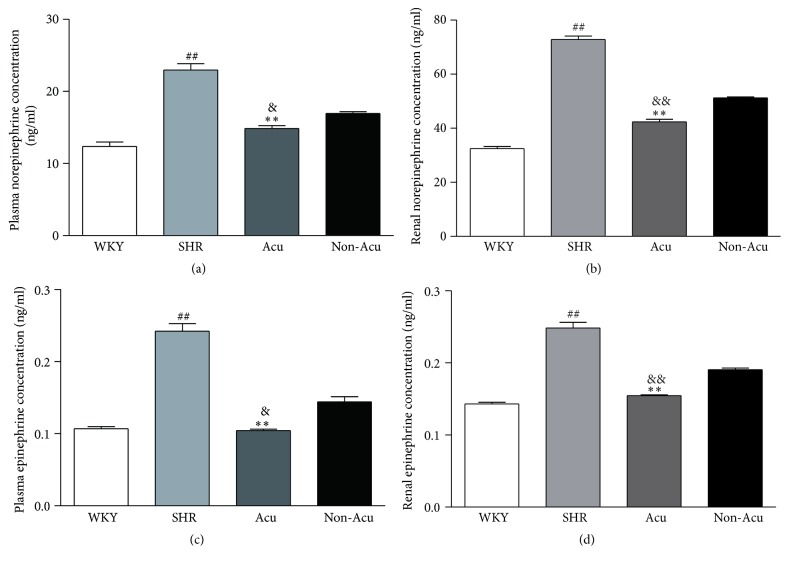
Effects of acupuncture on the contents of norepinephrine (a and b) and epinephrine (c and d) in plasma and renal tissues as measured by ELISA in all groups. Data are presented as mean ± SEM (*n* = 10 rats). ## indicate *P* < 0.01, compared with the WKY group; *∗∗* indicate *P* < 0.01, compared with SHR group; & and && indicate *P* < 0.05 and *P* < 0.01, respectively, compared with Non-Acu group.

The catecholamines, including norepinephrine (NE) and epinephrine (E), acting as neurotransmitters, play important roles in the sympathetic control of arterial BP. Therefore, we tested the contents of NE and E in plasma and renal tissues by ELISA (Figure 2). At the end of the study, NE and E levels induced by sympathetic nervous system activation were significantly higher in plasma and renal tissues from SHR than in those from WKY rats. Acupuncture at LR3 but not the nonacupoint significantly decreased NE and E concentrations in SHR group, and there was no significant difference between WKY group and Acu group.

### 3.4. Effect of Acupuncture on *β*-Adrenergic Receptor in SHRs

**Figure 3:**
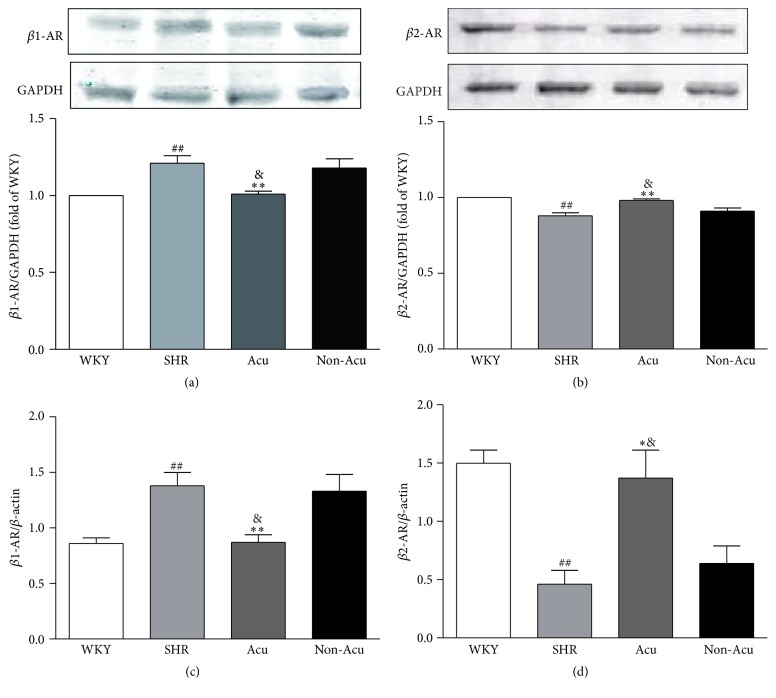
Effects of acupuncture on renal protein expression of *β*1-AR (a), *β*2-AR (b) and mRNA expression of *β*1-AR (c), *β*2-AR (d) in all groups. Data are presented as mean ± SEM (*n* = 10 rats). ## indicate *P* < 0.01, compared with the WKY group; *∗* and *∗∗* indicate *P* < 0.05 and *P* < 0.01, respectively, compared with SHR group; & indicate *P* < 0.05, compared with Non-Acu group.

Both NE and E act at G protein-coupled receptors of the adrenergic receptor family to mediate sympathetic effects. Within this main classification, there are several subtypes *α*1A, *α*1B, *α*1D, *α*2A, *α*2B, *α*2C, *β*1, *β*2, and *β*3. In this study we focused on two of these receptors, *β*1 and *β*2. Both of these receptor subtypes have been strongly implicated in cardiovascular control. To determine whether the antihypertensive effect of acupuncture is associated with beneficial outcome in *β*-ARs, we detected the mRNA and protein levels of *β*-ARs after acupuncture treatment by PCR and Western blot, respectively (Figure 3). Compared to WKY rats, SHR rats had significantly increased *β*1-AR protein levels and decreased *β*2-AR protein levels. After acupuncture, *β*1-AR protein levels were significantly decreased compared to SHR group. The signal ratio of *β*1-AR protein to actin was reduced by about 16.5% (1.01 versus 1.21). In contrast, *β*1-AR protein levels were similar between SHR group and Non-Acu group. The trends of the *β*-ARs mRNA levels were consistent with its protein levels.

### 3.5. Effect of Acupuncture on Plasma Renin Concentration, Renal Renin Content, and Renin mRNA Expression in SHRs

**Figure 4:**
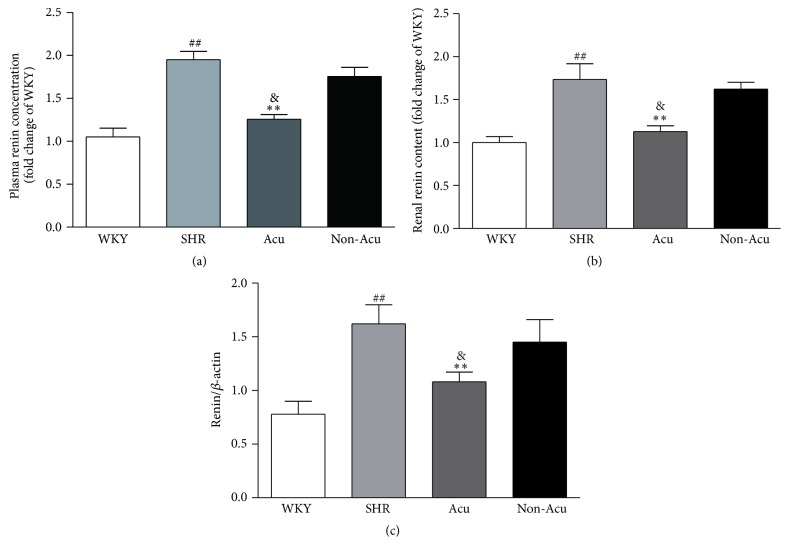
Effects of acupuncture on renin content (a and b) and renin mRNA expression (c). Data are presented as mean ± SEM (*n* = 10 rats). ## indicate *P* < 0.01, compared with the WKY group; *∗∗* indicate *P* < 0.01, compared with SHR group; & indicate *P* < 0.05, compared with Non-Acu group.

The juxtaglomerular (JG) cells in the media of renal afferent arterioles are the major sites of synthesis of the aspartic protease renin, the rate-limiting enzyme in the formation of angiotensin II. The JG cells are in contact with sympathetic nerve varicosities and express postjunctional *β*-ARs. Activation of *β*-ARs directly increases renin secretion. To address the role of acupuncture in the regulation of renin, we used radioimmunoassay to detect the concentration of renin. As shown in Figure 4, plasma renin concentration (PRC) increased significantly in SHR group and Non-Acu group and decreased significantly after acupuncture at LR3. Renal renin content was significantly higher in SHR rats compared with WKY rats. There was a significant reduction in renin content in the Acu group, whereas the decrease in renin content in Non-Acu group did not reach significance. Renal renin mRNA was markedly higher in SHR rats compared with WKY rats. Renin mRNA was significantly altered by acupuncture at LR3, whereas acupuncture at nonacupoint caused nonsignificant reductions of renin mRNA.

## 4. Discussion

In the present study, we show that acupuncture at LR3 reduced MBP and regulated HRV in SHR. And the therapeutic effects were associated with renal sympathetic nervous system, which can be manifested by the significant reductions in the levels of E and NE (an indirect marker of sympathetic activity) and the content of renin (indicative of increased renal sympathetic activity) in the plasma and renal by acupuncture. In addition, acupuncture decreased *β*1-AR expression and increased *β*2-AR expression, which means acupuncture has bidirectional regulation effects on *β*-ARs.

Autonomic imbalance with increased sympathetic activity has been strongly implicated in the pathophysiology of hypertension. Among the different available noninvasive techniques for assessing the autonomic status, HRV has emerged as a simple, noninvasive method to evaluate the sympathovagal balance. In this experiment, we focus on three HRV indexes. SDNN is associated with overall autonomic tone. Therefore, our findings of depressed SDNN in SHR rats suggested a homeostatic disruption in sympathovagal balance. However, because rMSSD is generally associated with vagal pathways, our results with unchanged rMSSD suggest an increase in sympathetic tone in SHR rats. This result is consistent with Schroeder's finding that individuals with low SDNN at baseline were at an increased risk of developing hypertension over 9 years of follow-up [21]. An analysis of LF/HF ratios rather than single components is considered by many investigators to better reflect the activity of the sympathovagal balance. Neto et al. showed a significant correlation between reduction BP levels and lower LF/HF ratio [22]. In our study, the higher LF/HF ratio demonstrated that SHRs have excessive sympathetic activation and autonomic imbalance. After acupuncture treatment, SDNN increased and LF/HF ratio decreased, but the rMSDD did not change. These results indicated that acupuncture could regulate the balance of the autonomic nervous system mainly through reducing the sympathetic activity.

Evidence collected throughout the years has shown that alterations in sympathetic cardiovascular control participate in the development, maintenance, and progression of hypertension. Renal sympathetic nerves induce high BP via E and NE regulates *β*-ARs expression. Of the two main *β*-AR subtypes (*β*1 and *β*2), *β*1-AR signaling has been linked to cardiotoxicity. In contrast, *β*2-AR activates signaling pathways involved in cardioprotection. Gui et al. found that *β*2-AR has a vasodilatation effect, and Raf kinase inhibitory protein could correct impaired *β*2-AR to treat hypertension [23]. Thus, some have proposed that the *β*1-AR is the “cardiotoxic subtype” whereas the *β*2-AR is the “cardioprotective subtype.” The two receptors regulate the peripheral resistance which reflects the balance between vasoconstrictor and vasodilator mechanisms. Based on this, we examined the effect of acupuncture on E, NE, and *β*-ARs. Here we observed that the contents of E and NE of SHR rats were significantly higher compared with the WKY rats. This gives direct evidence that the sympathetic nervous system is hyperreactive in the SHR and is in general agreement with previous studies [24, 25]. Then acupuncture at LR3, not nonacupoint, could decrease the contents of E and NE. In addition, acupuncture significantly decreased the expression of *β*1-AR and increased *β*2-AR expression in SHR. Therefore, the antihypertensive effect of acupuncture in SHR might be associated with the balance of *β*1/*β*2-AR. *β*-blockers therapy plays a major role in the treatment of cardiovascular diseases. To date, 14 *β*-blockers have received Federal Drug Administration approval for oral use in patients having systemic hypertension [26]. By receptor selective classification, *β*-blockers can be divided into three categories, namely, nonselective b-blocker, selective b1- receptor blocker, and b1-receptor blocker with additional *α*1-receptor blocking activity. Among them, the nonselective b-blocker is used most widely. In general, *β*-blockers are well tolerated, but serious side effects may occur, which are usually associated with the *β*2-AR antagonistic activity (e.g., increase in peripheral vascular resistance, worsening of asthma symptoms) [27]. Wong et al. suggested that *β*1 selective blockers lowered BP by a greater magnitude compared to dual receptor beta-blockers [28]. Acupuncture is reported to have potential for treating hypertension with fewer side effects [29]. Moreover, Zhang et al. reported that acupuncture may serve as an alternative for hypertensive patients, especially for those who cannot tolerate the side effects of antihypertensive drugs [30]. Combined with our experimental results, where acupuncture could decrease *β*1-AR expression and increase *β*2-AR expression, we consider that the low side effects of acupuncture may be related to the increased expression of *β*2 receptors, which needs to be further investigated.

The RAAS is an important mechanism in regulating BP. For that reason, an alteration in any molecule that composes the RAAS contributes to developing hypertension [31]. Classically, the sympathetic nervous system rapidly activates the RAAS through *β*1-AR mediated release of renin [32]. Therefore, inhibition of renin may exert complete inhibition of the RAAS, leading to a decrease of BP. Hisa et al. found that a *β*-AR blocker could reduce renal nerve stimulation-induced renin release. In our experiment, we found a significant reduction of renin content after acupuncture. Consistent with our results, Lohmeier et al. demonstrated that renal denervation decreased plasma renin content and abolished the hypertension [33]. However, the effect of acupuncture on RAAS in SHRs needs further study.

The renal sympathetic nervous system plays an active role in the modulation of BP, and its overactivation can lead to hypertension [34]. The Symplicity HTN-1 and HTN-2 studies proposed renal denervation as an effective and safe approach to treat patients with hypertension and were followed by substantial enthusiasm [35, 36]. In contrast with Symplicity HTN-1 and HTN-2, the announcement that Symplicity HTN-3 failed to meet its primary efficacy endpoint and put an abrupt stop to these overoptimistic expectations shows that renal denervation is not ready for clinical dissemination [37]. However, some researchers considered that the execution of the study was hampered by operational weaknesses and drug adherence [38, 39]. Despite the disappointing outcome of Simplicity HTN-3, the renal sympathetic system is still a potential target for treating hypertension, and our results indicate that acupuncture could reduce the renal sympathetic nervous activity and lower BP without related risk.

In conclusion, our results provide evidence that acupuncture at LR3 can significantly relieve the increased MBP through reducing renal sympathetic nervous activity. The effect of acupuncture on renal sympathetic nervous activity is evidenced by the improved HRV and the reduction of NE and E contents. The decreased *β*1-AR and increased *β*2-AR expression we observed may also be involved in the beneficial effect of acupuncture on hypertension.

## Figures and Tables

**Table 1:** Sequence for the primers and TaqMan probes for the rat *β*1-AR, *β*2-AR, and renin.

	Forward primer	Reverse primer
*β*1-AR	GCCGATCTGGTCATGGGAC	GGCGATGACACACAGGGTC
*β*2-AR	CATCCTCATGTCGGTTATC	ATGACTAGATCAGCACACG
Renin	GAGGCCTTCCTTGACCAATC	TGTGAATCCCACAAGCAAGG
